# Interaction of Sex and Diabetes on Outcome After Ischemic Stroke

**DOI:** 10.3389/fneur.2018.00250

**Published:** 2018-04-13

**Authors:** Maria Montserrat Soriano-Reixach, Rosa María Vivanco-Hidalgo, Angel Ois, Ana Rodríguez-Campello, Jaume Roquer

**Affiliations:** ^1^Universitat Pompeu Fabra and Universitat Autònoma de Barcelona, Barcelona, Spain; ^2^Servei de Neurologia, IMIM-Hospital del Mar, Barcelona, Spain; ^3^Departament de Medicina, Universitat Autònoma de Barcelona, Barcelona, Spain

**Keywords:** ischemic stroke, diabetes, sex differences, outcome, mortality

## Abstract

**Background:**

The relationship between ischemic stroke (IS), diabetes mellitus (DM), and sex is intriguing. The aim of this study was to assess the effect modification of sex in the association between DM and short- and long-term disability and mortality in first-ever IS patients.

**Methods:**

In a retrospective, observational, hospital-based study of a prospective series including first-ever IS patients from January 2006 until July 2011, differences in 3-month and 5-year mortality, and disability between diabetic and non-diabetic patients [modified Rankin Scale (mRS) from 3 to 5] were analyzed by sex.

**Results:**

In total, 933 patients (36.3% with DM, 50.5% women) were included. Overall 3-month and 5-year mortality were 150 (16.1%) and 407 (44.1%), respectively. Adjusted for age, previous mRS, and stroke severity, patients with DM had significantly higher 3-month disability [hazard ratio (HR): 1.49 (95% confidence interval (CI): 1.39–1.70), *p* < 0.0001], 5-year disability [HR: 1.41 (95% CI: 1.07–1.86), *p* = 0.015], and 5-year mortality [HR: 1.48 (95% CI: 1.20–1.81), *p* < 0.0001], compared with the non-DM group. Compared with non-DM women, women with diabetes had worse 3-month disability [HR: 1.81 (95% CI: 1.33–2.46), *p* < 0.0001] and 5-year mortality [HR: 1.72 (95% CI: 1.30–2.20), *p* < 0.0001], and a trend for 5-year disability [HR: 1.40 (95% CI: 0.99–2.09), *p* = 0.057]. In men, DM had an effect on 3-month disability [HR: 1.45 (95% CI: 1.07–1.96), *p* = 0.018], a trend for 5-year disability [HR: 1.43 (95% CI: 0.94–2.19), *p* = 0.096], but no clear effect on 5-year mortality [HR: 1.22 (95% CI: 0.91–1.65), *p* = 0.186].

**Conclusion:**

Sex has a modifier effect on mortality in first-ever IS diabetic patients. Long-term mortality is increased in diabetic women compared with non-diabetic women, a difference not observed in men.

## Introduction

Diabetes mellitus (DM) is an independent risk factor for stroke and cardiovascular disease (1). Patients with DM have up to threefold increased risk of recurrent stroke, compared with those with normal glucose levels (2, 3). Additionally, DM raises ischemic stroke (IS) severity, and has been associated with a poor functional outcome, worse long-term vascular prognosis, and increased mortality after stroke onset, compared with non-diabetic patients (4–6). Some studies have associated glycemic values with the poor short-term outcome, concluding that high-glycemia levels during acute IS, but not DM diagnosis, increased the odds for poor 30-day to 3-month outcome (7–9).

The relationship between IS, DM, and sex is intriguing. A recent meta-analysis has estimated that women with DM have a 27% greater risk of stroke than men with DM (10), and some studies suggest that they have worse survival and functional outcome than men (11–14). Other studies, however, have found increased mortality for both women and men with diabetes (15), a time-dependent mortality risk only for DM men (16), and lower adjusted mortality in DM women than in men (17) The reasons for these sex differences are unknown, and the majority of the research has focused on comparing men and women with DM, with little information about how sex can influence the short- and long-term impact of DM on post-IS prognosis. On the other hand, many of these studies are unadjusted (12, 17) or adjusted only by age and vascular risk factors (14–17) or previous treatments (15, 16) without taking into account two of the most important predictors of mortality: previous disability and stroke severity.

The aim of this study was to assess the effect modification of sex in the association between DM and short- and long-term disability and mortality in first-ever IS patients, as a primary end-point. We also sought to analyze, as a secondary end-point, whether differences exist between DM and non-DM patients in acute stroke management and cardiovascular events during 5-year follow-up.

## Materials and Methods

The study was a retrospective analysis of a prospective hospital-based stroke register. From January 2006 through July 2011, 1,542 patients with a first-ever IS were admitted to our hospital. Patients with previous modified Rankin Scale (mRS) >3, transient ischemic attack, missing follow-up data, inaccurate or incomplete clinical information, unusual cause of stroke, residence outside our hospital reference area, or those who refused to participate in the BASICMAR database (*n* = 8) were excluded. The BASICMAR database (18) is an ongoing prospective register of patients with acute stroke at University Hospital del Mar, a tertiary public hospital serving a population of 330,000 in three districts of the city of Barcelona. The final cohort was 933 patients.

All included patients received a computed tomography (CT) scan in the emergency room and were evaluated at hospital admission by a vascular-trained neurologist who established initial severity using the National Institute of Health Stroke Scale (NIHSS). Stroke subtype was categorized using the TOAST classification (19). Additional CT, MRI evaluations, or angiographic studies were done, as needed, during hospitalization. HbA1c was measured during hospital admission in 300 of 339 diabetic patients (88.5%).

Vascular risk factors, as defined by international guidelines, were obtained from the patient, relatives, caregivers, or previous medical records after stroke onset. A structured questionnaire was used to record the following variables: arterial hypertension (evidence of at least two raised blood pressure measurements, systolic >140 mmHg or diastolic >90 mmHg, recorded on different days before stroke onset; a physician’s diagnosis; or use of medication); diabetes (previous physician diagnosis or use of medication); hyperlipidemia (physician diagnosis, use of medication, serum cholesterol concentration >220 mg/dL, low-density lipoprotein cholesterol >130 mg/dL, or serum triglyceride concentration >150 mg/dL); ischemic heart disease (IHD) (documented history of angina pectoris or myocardial infarction); peripheral arterial disease (PAD) (documented current intermittent claudication with an ankle brachial index <0.9 or a history of intermittent claudication, together with a previous related intervention, such as amputation); atrial fibrillation (AF) (physician diagnosis, use of medication, or conclusive electrocardiogram data); current smoking habit; alcohol overuse (>60 g/day); and illicit drug use. Atherosclerosis burden was calculated as previously described (20), assigning a score of 0 to patients with no previous diagnosis of IHD or PAD, 1 to a diagnosis of IHD or PAD, and 2 to patients with the concomitant presence of both diseases. For DM patients, the usual pre-stroke treatment for DM was recorded.

Under national and international guidelines, treatment included recombinant tissue plasminogen activator (rtPA) (first 4.5 h) and endovascular therapy (21). During hospitalization, additional glucose testing and HbA1C were done by systematic protocol, when indicated; new DM cases were determined at discharge.

The primary end-points were mortality and functional outcome at 3-month and 5-year follow-up. The secondary end-points were: (a) acute stroke care (admission to a monitored acute care stroke unit, intravenous rtPA treatment or endovascular thombectomy) and (b) new vascular events, including stroke, intracranial hemorrhage, coronary artery disease (CAD), and cardiovascular death, recorded at 5-year follow-up, along with the detection of any previously unknown AF. Mortality and disability data, vascular recurrences, and the detection of previously non-diagnosed AF were obtained from a clinic visit at 3-month and 5-year follow-up, from electronic medical records, hospital admissions records, or by telephone contact with primary care physicians.

### Statistical Analysis

Age and NIHSS score had a non-normal distribution and were expressed as medians and interquartile ranges (IQR). Categorical data were expressed as counts and percentages. Differences in parametric and non-parametric continuous variables were evaluated using the *t*-test and Mann–Whitney *U* test, respectively, and the χ^2^ test was used for proportional analysis. We compared by bivariate analysis the demographic, vascular risk, and stroke care differences between: (1) DM and non-DM patients; (2) DM women vs non-DM women, and (3) DM men vs non-DM men.

Three-month and 5-year disability and mortality were compared with test differences in prognosis between DM and non-DM patients, and stratified by sex. To analyze the interaction of sex and diabetes, bivariate, and Cox regression analyses adjusted for age, previous mRS, and NIHSS score at admission were performed for both men and women to assess 3-month and 5-year disability and 5-year mortality by sex. The effect modification of sex was tested using interactions (diabetes-sex) in the models. For the secondary end-points, we compared the number of patients admitted to monitored acute care stroke unit and the number of patients treated with intravenous rtPA or mechanical thrombectomy between diabetic and non-diabetic women, and diabetic and non-diabetic men.

The hazard ratios (HRs) were presented with 95% confidence intervals (CIs). The significance level was set at 0.05. All analyses were two-tailed and performed with the SPSS statistical package or Stata 12 package.

Standard Protocol Approvals, Registrations, and Patient Consents: The information used in this study was collected from the prospective BASICMAR register, with the approval of our local ethics committee (CEIC-Parc de Salut Mar). All patients gave their informed consent prior to their inclusion in the study.

## Results

A total of 933 patients were included in the analysis. We excluded 609 patients for the following reasons, according to the established criteria: previous poor functional status (mRS > 3), 102; transient ischemic attack, 206; missing follow-up data, 19; inaccurate or incomplete clinical information, 29; unusual cause of stroke, 42; residence outside our hospital reference area, 203; and declined participation in the BASICMAR database, 8. There was no difference between the excluded and included patients in the percentage of DM cases (9.7 vs 9.8%, respectively). However, excluded patients were older [82.7 (9.5) vs 72.9 (12.6) years, *p* < 0.001], more likely to be women (13.5 vs 6.3%, *p* < 0.0001), and had more severe strokes (*p* > 0.0001), AF (*p* = 0.002), and IHD (*p* = 0.001), compared with included patients. Baseline characteristics of the study population are shown in Table 1. Missing data included history of PAD (*n* = 2), arterial hypertension (*n* = 3), hyperlipidemia (*n* = 5), atherosclerotic burden (*n* = 6), data on stroke unit admission (*n* = 9), smoking (*n* = 18), overuse of alcohol (*n* = 20), new cardiovascular events during follow-up (*n* = 21), new AF during follow-up (*n* = 28), 5-year mRS (*n* = 85), and mortality (*n* = 10). Patients with missing follow-up data were older than those with complete data [75.0 (12.1) vs 70.0 (11.6) years, *p* < 0.0001]; there were no differences in sex, previous mRS, risk factors (hypertension, DM, hyperlipidemia, AF, PAD, CAD, smoking, and alcohol intake) and stroke severity.

**Table 1:** Differences between diabetic and non-diabetic patients.

	Total (*n* = 933)	DM (*n* = 339)	Non-DM (*n* = 594)	Unadjusted HR (95% CI)	*p*-Value	Adjusted^a^ HR (95% CI)	*p*-Value
**Demographic and vascular risk factors**							
Age, years, median (IQR)	77 (68–83)	76 (68–82)	78 (68–84)		0.056		
Sex, female, *n* (%)	471 (50.5)	162 (47.8)	309 (52.0)		0.221		
Hypertension, *n* (%)	669 (71.9)	269 (79.6)	400 (67.6)	1.87 (1.37–2.57)	0.0001		
Hyperlipidemia, *n* (%)	387 (41.7)	174 (51.8)	213 (36.0)	1.91 (1.46–2.51)	0.0001		
Atrial fibrillation, *n* (%)	329 (35.3)	117 (34.5)	212 (35.7)		0.722		
Coronary artery disease, *n* (%)	143 (15.4)	72 (21.4)	61 (12.0)	1.99 (1.39–2.86)	0.0001		
Peripheral arterial disease, *n* (%)	79 (8.5)	40 (11.9)	39 (6.6)	1.92 (1.21–3.05)	0.007		
Smoking, *n* (%)	184 (20.1)	55 (16.6)	129 (22.1)	0.70 (0.49–0.99)	0.048		
Alcohol overuse, *n* (%)	168 (18.4)	61 (18.5)	107 (18.4)		1.0		
Previous disability, median (IQR)	0(0–0)	0 (0–1)	0 (0–0)		0.0001		

**Stroke characteristic and acute treatment data**							
NIHSS points, median (IQR)	5 (3–13)	5 (3–11)	6 (3–14)		0.070		
Atherothrombotic stroke, *n* (%)	131 (14.9)	61 (18.0)	70 (11.8)				
Lacunar stroke, *n* (%)	217 (23.3)	79 (23.3)	138 (23.2)		0.047		
Cardioembolic stroke, *n* (%)	345 (37.0)	122 (36.0)	223 (37.5)				
Stroke of undetermined cause^b^, *n* (%)	240 (25.7)	77 (22.7)	163 (27.4)				
NIHSS points, median (IQR)	5 (3–13)	5 (3–11)	6 (3–14)		0.070		
rtPA treatment	162	45 (13.3)	117(19.7)	0.62 (0.43–0.91)	0.015	1.01 (0.71–1.44)	0.963
Endovascular or rtPA treatment	175	50 (14.7)	125 (21.0)	0.65 (0.45–0.939)	0.019	1.06 (0.75–1.48)	0.756

**Outcome measures**							
New atrial fibrillation^c^, *n* (%)	58 (10.1)	13 (6.0)	45 (12.5)	0.47 (0.25–0.89)	0.017	0.62 (0.33–1.16)	0.135
Total cardiovascular events, *n* (%)	290 (31.8)	124 (37.3)	166 (28.6)	1.49 (1.12–1.98)	0.006	1.50 (1.18–1.90)	0.001
Stroke recurrence, *n* (%)	141(15.5)	57 (17.2)	84 (14.5)	1.22 (0.85–1.77)	0.280		
Coronary artery disease, *n* (%)	47 (5.2)	20 (6.0)	27 (4.7)	1.31 (0.72–2.38)	0.368		
Peripheral arterial disease, *n* (%)	64 (7.0)	31 (9.3)	33 (5,7)	1.71 (1.03–2.84)	0.043	1.76 (1.07–2.90)	0.026
Intracranial hemorrhage, *n* (%)	19 (2.1)	7 (2.1)	12 (2.1)	1.02 (0.40–2.62)	0.968		
Cardiovascular death, *n* (%)	27 (3.0)	14 (4.2)	13 (2.2)	1.92 (0.89–4.13)	0.091	2.19 (1.01–4.72)	0.046
3-month mortality, *n* (%)	150 (16.1)	55 (16.2)	95 (16.0)	1.02 (0.71–1.46)	0.926	1.13 (0.81–1.58)	0.479
3-month disability, *n* (%)	360 (43.0)	151 (50.0)	209 (39.0)	1.57 (1.18–2.08)	0.002	1.49 (1.39–1.70)	0.0001
5-year mortality, *n* (%)	407 (43.6)	170 (50.7)	237 (40.4)	1.52 (1.16–1.99)	0.002	1.48 (1.20–1.81)	0.0001
5-year disability, *n* (%)	219 (50.5)	89 (61.0)	130 (45.1)	1.90 (1.27–2.85)	0.002	1.41 (1.07–1.86)	0.015

*^a^Adjusted for age, previous functional disability (by modified Rankin Scale), and stroke severity (by National Institute of Health Stroke Scale)*.

*^b^Stroke of undetermined cause includes unknown cause, more than one possible cause, or insufficient study*.

*^c^New atrial fibrillation = diagnoses of atrial fibrillation during 5-year follow-up*.

Overall, 339 (36.3%) patients had DM. HbA1c during admission was available in 300/339 (88.5%) diabetic patients, 145/162 (89.5%) women, and 155/177 (87.6%) men. Median HbA1c (IQR) was 7.1 (6.5–8.2) for diabetic women and 7.5 (6.6–9.0) for diabetic men, *p* = 0.051. Regarding diabetes treatment previous to stroke, 186 (54.9%) patients were taking oral antidiabetics [88/162 women (54.3%), and 98/177 (55.4%) men, *p* = 0.847; and 75 (22.1%) were under insulin treatment, 39/162 women (24.1%) and 36/177 men (20.3%), *p* = 0.408].

Table 1 shows that patients with DM had more vascular risk factors, worse previous functional status, and received less rtPA treatment than non-DM patients. Atherothrombotic stroke was more frequent in DM (18.0%) than in non-DM patients (11.8%), *p* = 0.047. During the 5-year follow-up, DM patients were more likely to have a cardiovascular event than non-DM patients (HR: 1.42, 95% CI: 1.06–1.91; adjusted *p* = 0.019), with PAD being the only event subtype that differed significantly between the two groups (HR: 1.81, 95% CI: 1.18–2.76; adjusted *p* = 0.006). In contrast, new AF was detected more frequently in non-DM than in DM patients (*p* = 0.026). At 3 months, there were no mortality differences between DM and non-DM patients (adjusted *p* = 0.466). However, unadjusted and adjusted 3-month, and 5-year disability, and 5-year mortality, were greater in DM than in non-DM patients (adjusted HR: 1.49, 95% CI: 1.39–1.70, *p* < 0.0001; adjusted HR: 1.41, 95% CI: 1.07–1.86, *p* < 0.015; adjusted HR: 1.48, 95% CI: 1.20–1.81, *p* < 0.0001; respectively).

Table 2 shows the differences between DM and non-DM women and DM and non-DM men. Diabetic women had more vascular risk factors, more previous disability, more atherosclerotic burden, and less current smoking than non-DM women. There were no differences in stroke etiology between DM and non-DM women (*p* = 0.830). Men with diabetes had more vascular risk factors than non-diabetic patients in general, and were less likely to receive acute reperfusion treatments. After adjustment, however, this difference disappeared. There were more atherothrombotic strokes (25.4%) and fewer strokes with undetermined cause (19.8%) in DM men compared with non-DM men (15.9 and 29.8%, respectively, *p* = 0.030).

**Table 2:** Differences between diabetic and non-diabetic women and diabetic and non-diabetic men.

	Total	DM	Non-DM	Unadjusted HR (95% CI)	*p*-Value	Adjusted^a^ HR (95% CI)	*p*-Value
Women	(*n* = 471)	(*n* = 162)	(*n* = 309)				

**Demographic and vascular risk factors**							
Age, years, median (IQR)	80 (73–85)	79 (73–84)	80 (73–86)		0.635		
Hypertension, *n* (%)	369 (78.7)	137 (85.1)	232 (75.3)	1.87 (1.13–3.10)	0.017		
Hyperlipidemia, *n* (%)	201 (42.9)	89 (54.9)	112 (36.5)	2.12 (1.44–3.13)	0.0001		
Atrial fibrillation, *n* (%)	225 (46.8)	81 (50.0)	144 (46.6)	1.14 (0.78–1.68)	0.498		
Coronary artery disease, *n* (%)	62 (13.2)	36 (22.4)	26 (8.4)	3.12 (1.81–5.40)	0.0001		
Peripheral arterial disease, *n* (%)	20 (4.3)	8 (5.09)	12 (3.9)	1.29 (0.52–3.23)	0.632		
Smoking, *n* (%)	30 (6.5)	5 (3.1)	25 (8.3)	0.35 (0.13–0.94)	0.030		
Alcohol overuse, *n* (%)	26 (5.6)	10 (6.3)	16 (5.3)	1.19 (0.53–2.68)	0.676		
Previous disability, median (IQR)	0 (0–2)	0 (0–1)	0 (0–0)		0.0001		
Atherosclerotic burden, median (IQR)	0 (0–0)	0 (0–1)	0 (0–0)		0.0001		

**Stroke characteristic and acute treatment data**							
NIHSS points, median (IQR)	7 (4–16)	7 (3–17)	7 (4–16)		0.963		
Atherothrombotic stroke, *n* (%)	41 (8.7)	16 (9.9)	25 (8.1)				
Lacunar stroke, *n* (%)	75 (15.9)	23 (14.2)	52 (16.8)		0.830		
Cardioembolic stroke, *n* (%)	232 (49.3)	81 (50.0)	151 (48.9)				
Stroke of undetermined cause, *n* (%)	123 (26.1)	42 (25.9)	81 (26.2)				
rtPA treatment	85 (18.0)	25 (15.4)	60 (19.4)	0.76 (0.45–1.26)	0.315		
rtPA/endovascular treatment, *n* (%)	95 (20.2)	28 (17.3)	67 (21.7)	0.76 (0.46–1.23)	0.279		
Stroke unit admission, *n* (%)	390 (83.3)	128 (79.5)	262 (85.3)	0.67 (0.41–1.09)	0.118		

**Outcome measures**							
New atrial fibrillation^b^, *n* (%)	34 (7.3)	7 (4.3)	27 (8.9)	0.47 (0.20–1.09)	0.092		
Total cardiovascular events, *n* (%)	128 (27.6)	51 (32.1)	77 (25.3)	1.39 (0.91–2.12)	0.127		
Stroke recurrence, *n* (%)	67 (14.5)	27 (17.0)	40 (13.2)	1.35 (0.79–2.30)	0.267		
Coronary artery disease, *n* (%)	19 (4.1)	10 (6.3)	9 (3.0)	2.20 (0.88–5.53)	0.086		
Peripheral arterial disease, *n* (%)	26 (5.6)	11 (6.9)	15 (4.9)	1.43 (0.64–3.20)	0.399	1.63 (073–3.64)	0.235
Intracranial hemorrhage, *n* (%)	9 (1.9)	2 (1.3)	7 (2.3)	0.54 (0.11–2.63)	0.439		
Cardiovascular death, *n* (%)	13 (2.8)	8 (5.0)	5 (1.7)	3.15 (1.02–9.82)	0.037	3.76 (1.18–12.0)	0.025

Men	(*n* = 462)	(*n* = 177)	(*n* = 285)				

**Demographic and vascular risk factors**							
Age, years, median (IQR)	73 (64–81)	72 (64–79)	75 (64–82)		0.083		
Hypertension, *n* (%)	300 (65.1)	132 (74.6)	168 (59.2)	2.03 (1.34–3.06)	0.001		
Hyperlipidemia, *n* (%)	186 (40.5)	85 (48.9)	101 (35.4)	1.74 (1.19–2.55)	0.006		
Atrial fibrillation, *n* (%)	104 (22.5)	36 (20.3)	68 (23.9)	0.82 (0.52–1.29)	0.423		
Coronary artery disease, *n* (%)	81 (17.6)	36 (20.5)	45 (15.8)	1.37 (0.84–2.22)	0.211		
Peripheral arterial disease, *n* (%)	59 (12.8)	32 (18.2)	27 (9.5)	2.12 (1.22–3.69)	0.009		
Smoking, *n* (%)	154 (33.9)	50 (29.2)	104 (36.7)	0.71 (0.47–1.07)	0.125		
Alcohol overuse, *n* (%)	142 (31.4)	51 (30.0)	91 (32.3)	0.90 (0.60–1.36)	0.676		
Previous disability, median (IQR)	0 (0–0)	0 (0–1)	0 (0–0)		0.112		
Atherosclerotic burden, median (IQR)	0 (0–1)	0 (0–1)	0 (0–0)		0.009		

**Stroke characteristic and acute treatment data**							
NIHSS points, median (IQR)	5 (2–9)	4 (2–6)	5 (2–12)		0.068		
Atherothrombotic stroke, *n* (%)	90 (19.5)	45 (25.4)	45 (15.9)				
Lacunar stroke, *n* (%)	142 (30.7)	56 (31.6)	86 (30.2)				
Cardioembolic stroke, *n* (%)	113 (24.5)	41 (23.2)	72 (25.3)		0.030		
Stroke of undetermined cause^c^, *n* (%)	117 (25.3)	35 (19.8)	82 (29.8)				
rtPA treatment	77 (16.7)	20 (11.3)	57 (20.0)	0.51 (0.29–0.88)	0.015		
rtPA/endovascular treatment, *n* (%)	81 (17.5)	22 (12.4)	95 (20.7)	0.54 (0.32–0.92)	0.024		
Stroke unit admission, *n* (%)	402 (88.2)	154 (88.0)	248 (88.3)	0.98 (0.55–1.75)	1		

**Outcome measures**							
New atrial fibrillation^b^, *n* (%)	24 (5.5)	6 (3.5)	18 (6.8)	0.50 (0.19–1.28)	0.197		
Total cardiovascular events, *n* (%)	162 (36.1)	73 (42.2)	89 (32.2)	1.39 (0.91–2.12)	0.034	1.34 (0.98–1.83)	0.065
Stroke recurrence, *n* (%)	74 (16.5)	30 (17.3)	44 (15.9)	1.11 (0.67–1.84)	0.697		
Coronary artery disease, *n* (%)	28 (6.2)	10 (5.8)	18 (6.5)	0.88 (0.40–1.95)	0.752		
Peripheral arterial disease, *n* (%)	38 (8.5)	20 (11.6)	18 (6.5)	1.87 (0.96–3.65)	0.081	1.73 (0.91–2.29)	0.093
Intracranial hemorrhage, *n* (%)	10 (2.2)	5 (2.9)	5 (1.8)	1.61 (0.46–5.66)	0.518		
Cardiovascular death, *n* (%)	14 (3.1)	6 (3.5)	8 (2.9)	1.20 (0.41–3.53)	0.784		

*^a^Adjusted for age, previous functional disability (by modified Rankin Scale), and stroke severity (by National Institute of Health Stroke Scale)*.

*^b^New atrial fibrillation (AF) = diagnoses of AF during 5-year follow-up*.

*^c^Stroke of undetermined cause includes unknown cause, more than one possible cause, or insufficient study*.

### Primary End-Points

Women with diabetes showed higher 3-month disability (adjusted HR: 1.81, 95% CI: 1.33–2.46, *p* < 0.0001), and 5-year mortality (adjusted HR: 1.72, 95% CI: 1.30–2.20, *p* < 0.0001), and a trend for 5-year disability (adjusted HR: 1.40, 95% CI: 0.99–2.09, *p* = 0.057), compared with non-DM women (Table 3). In men, DM had an effect on 3-month disability (adjusted HR: 1.45, 95% CI: 1.07–1.96, *p* = 0.018), a trend for 5-year disability (adjusted HR: 1.43, 95% CI: 0.94–2.19, *p* = 0.096), but no effect on 5-year mortality (adjusted HR: 1.22, 95% CI: 0.91–1.65, *p* = 0.186). Table 3 also shows the results of the interaction analyses between DM and sex.

**Table 3:** Outcome parameters comparing diabetic women with non-diabetic women and diabetic men with non-diabetic men.

	DM	Non-DM	Unadjusted HR (95% CI)	*p*-Value	Adjusted^a^ HR (95% CI)	*p*-Value
**Women**						
3-month disability, *n* (%)	77 (61.6)	103 (41.0)	2.31 (1.49–3.58)	0.0001	1.81 (1.33–2.46)	0.0001
5-year disability, *n* (%)	48 (78.7)	83 (54.6)	3.07 (1.54–6.13)	0.001	1.40 (0.99–2.09)	0.057
5-year mortality, *n* (%)	96 (59.3)	121 (39.4)	2.24 (1.52–3.30)	0.0001	1.72 (1.30–2.20)	0.0001

**Men**						
3-month disability, *n* (%)	74 (41.8.2)	106 (37.2)	1.21 (0.83–1.78)	0.323	1.45 (1.07–1.96)	0.018
5-year disability, *n* (%)	41 (48.2)	47 (34.6)	1.77 (1.02–3.07)	0.049	1.43 (0.94–2.19)	0.096
5-year mortality, *n* (%)	74 (42.8)	116 (41.4)	1.06 (0.72–1.55)	0.845	1.22 (0.91–1.65)	0.186

**DM:sex interaction^b^**						
3-month disability				0.0186		
5-year disability				0.0049		
5-year mortality						0.0001

*^a^Adjusted by age, previous functional disability (by modified Rankin Scale), and stroke severity (by National Institute of Health Stroke Scale)*.

*^b^p-Values obtained through Wald Test*.

**Figure 1:**
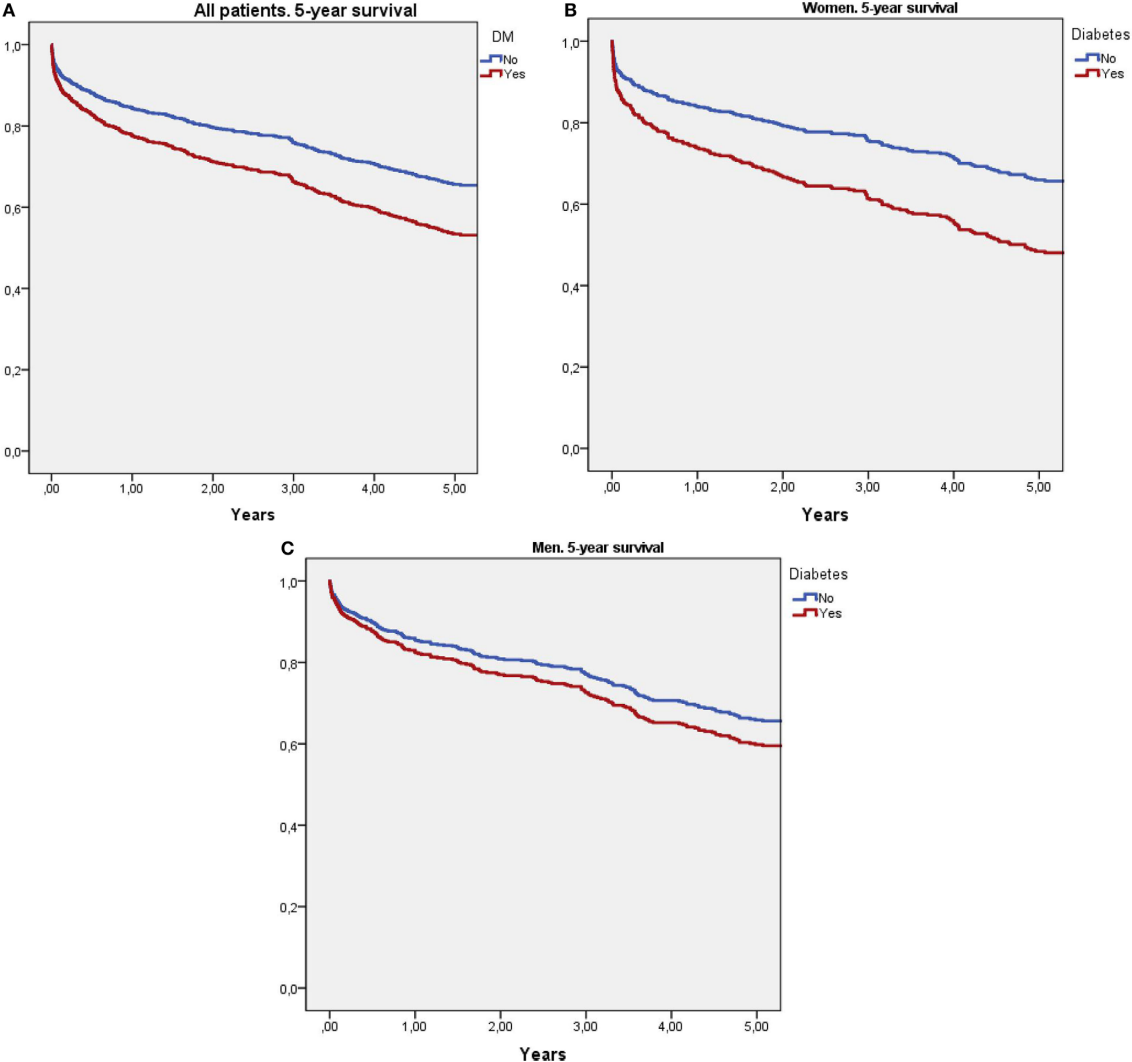
Survival curves using Cox regression analysis adjusted for age, stroke severity (National Institute of Health Stroke Scale), and previous modified Rankin Scale score. **(A)** Compares 5-year survival between diabetic and non-diabetic patients. **(B)** Compares 5-year survival between diabetic and non-diabetic women. **(C)** Compares 5-year survival between diabetic and non-diabetic men.

Cox survival curves adjusted for age, stroke severity (NIHSS), and previous mRS are shown in Figure 1. Figure 1A shows that patients with diabetes had higher 5-year mortality than non-DM patients (HR: 1.49, 95% CI: 1.22–1.82, *p* < 0.0001). Figure 1B shows that women with diabetes had significantly higher 5-year mortality than non-DM women (HR: 1.74, 95% CI: 1.32–2.31, *p* < 0.0001). Figure 1B shows that in men, DM had no effect on 5-year mortality (HR: 1.23, 95% CI: 0.91–1.66, *p* = 0.173).

### Secondary End-Points

No differences were observed between women with and without DM or between men with and without DM in the variables related to acute stroke care: stroke unit admission, intravenous rtPA treatment, and endovascular thrombectomy (Table 2). Regarding cardiovascular events during follow-up, although no difference in total cardiovascular events was observed, women with DM had higher cardiovascular-specific mortality, compared with non-DM women (adjusted HR: 3.76, 95% CI: 1.18–12.0, *p* = 0.025). For men, there was a trend of higher cardiovascular events (HR: 1.34, 95% CI: 0.98–1.83, *p* = 0.065) and a trend of higher new PAD during follow-up in DM compared with non-DM (adjusted HR: 1.73, 95% CI: 0.91–2.29, *p* = 0.093).

## Discussion

The main finding of our study was that diabetes has a sex-related modifier effect on IS outcomes: women with DM had higher 5-year mortality compared with women without diabetes, but this effect was not significant in men.

The demographic differences between diabetic and non-diabetic patients observed in our study are consistent with the literature (13, 22, 23). Diabetic patients were older and had more vascular risk factors, such as hypertension, hyperlipidemia, coronary, and PADs, and worse previous functional status. Regarding the interaction of sex and diabetes, women with diabetes had more vascular risk factors, more previous disability, more atherosclerotic burden, less current smoking, and higher cardiovascular-specific mortality, compared with non-DM women. Men with diabetes had more vascular risk factors and a trend of new cardiovascular events, especially new PAD during follow-up, compared with non-diabetic patients. As in some previous studies, we observed no DM effect on short-term mortality (6, 24, 25); however, DM was an independent risk factor for long-term mortality for the whole series (before sex interaction analyses). Moreover, diabetic stroke survivors had greater probability of disability at 3 months and at 5 years, compared with non-DM patients, results similar to some previous reports (25). In our analysis, we found that patients with diabetes had a 5-year cumulative cardiovascular event rate of 37.3%, higher than in non-diabetics. The 5-year stroke recurrence rate in DM patients was 17.2%, similar to a previous meta-analysis (26).

Regarding the primary end-points of the study, the differences in the outcome measures between women with and without DM and men with and without DM support a sex-related modifier effect on IS outcomes in DM patients suggesting a different role of DM in men, compared with women. There was a special effect on long-term mortality, where DM had a deleterious effect in women but only a slight trend in men. Our study was adjusted for the three most powerful variables associated with stroke death: age, previous disability, and stroke severity. This reinforces previous studies showing deleterious effects of DM in women with IS (11–14). Our results differ from a German IS study that found no DM-related mortality differences for women but diabetic men with IS had lower short-term mortality, compared with non-diabetic men; notably, this effect did not persist after a year (16). A recent Canadian study showed lower mortality in DM women (17). However, neither study adjusted for stroke severity and disability previous to the index stroke.

The reason for the greater deleterious effect of DM on outcome we observed in women, compared with men, is unknown. It is well accepted that women have worse outcome than men after IS (27). Some researchers have reported that women are in worse condition than men at the time of new DM diagnosis, presenting with greater endothelial dysfunction and more severe hypertension due to underdetection and undertreatment of diabetes (10, 28). The inflammatory effect of diabetes has been established, along with its greater importance as a risk factor for stroke in women (3, 29, 30) and aggravates brain damage from stroke (31), compared with men. Other risk factors mainly affecting women have been described, such as relative longevity and sex-specific immune response and hormones (32, 33). Lower estrogen levels in postmenopausal women reduce the anti-inflammatory and neuroprotective effects of the hormone (34). Therefore, the high-inflammatory environment produced by diabetes, combined with lower neuroprotection from estrogens, may aggravate brain damage, and consequently increase morbidity and mortality outcomes in women with diabetes. Further studies in this direction might help to reveal the etiology of these differences in outcomes.

Regarding the study’s secondary end-point, and in order to search for a mechanism that could explain the higher mortality in DM than non-DM patients, we analyzed whether differences in acute care or acute treatment between these patient groups exist, with negative result. We also did not find higher stroke recurrence in the DM group, but a significant increase in cardiovascular death was observed, compared with non-DM women. Again, this could suggest a specific, sex-related, deleterious effect of diabetes for women with IS. However, the fact that we had not found these differences in DM compared with non-DM men, also raises the question of other factors more related to gender that could have had influence in the results (i.e., chronic conditions such as diabetes are differently addressed in women than men by physicians, and have a different self-management impact) (35). Regarding the controversy about whether women have worse DM control than men that could explain a worse outcome in DM patients (36, 37), our study analyzed DM control (by HbA1c levels previous to IS), finding a non-significant trend for a better control in women than in men (*p* = 0.051). In our study, patients with high-previous disability were excluded (102 cases, 69 women, and 33 men) because one of our aims was to analyze the effect of DM on functional outcome after IS. We believe that the eventual impact on the end-points of the study due to the exclusion of these cases is small, because the percentage of DM patients was quite similar between excluded women and men (34.8 vs 36.4%, *p* = 0.827) and between all cases excluded due to previous disability and all included patients (35.3 vs 36.3%, *p* = 0.739).

### Limitations

The present study has several limitations. The first potential bias is due to the single-center design. However, stroke care in Catalonia is very homogeneous, and the organization of the stroke code ensures that the vast majority of acute IS cases in our catchment area are referred to our hospital with no bias. Moreover, cases from other healthcare service areas were excluded. Second, cardiovascular recurrences and new AF diagnoses were assessed retrospectively. However, as all patients were from our catchment area, in most cases we could review their medical reports or contact the patient or medical practitioner. Third, the mRS was used to evaluate disability but, in some cases, this was done by telephone or by examining medical reports. Fourth, we did not have complete data on all causes of death during the 5-year follow-up. Our study also has strengths, because the cohort was quite large and well characterized, with complete baseline clinical data and follow-up that, despite being retrospective, was done with minimal losses to follow-up.

## Conclusion

Our study showed that diabetes remarkably increases the probability of both death and disability in IS patients, and supported a sex-related modifier effect on IS outcomes, as the worst outcome was observed only in women with diabetes. The explanation of this greater deleterious effect of diabetes on women with IS remains to be elucidated.

## Ethics Statement

The information used in this study was collected from the prospective BASICMAR register, with the approval of our local ethics committee (CEIC-Parc de Salut Mar). All patients gave their informed consent prior to their inclusion in the study.

## Author Contributions

JR designed the study; MS-R, RV-H, AO, AR-C, and JR analyzed data; MS-R, and JR wrote the manuscript; RV-H, AO, and AR-C contributed to data interpretation and revising the manuscript.

## Conflict of Interest Statement

The authors declare that there is no conflict of interest associated with this manuscript. Publication is approved by all authors and explicitly by the responsible authorities where the work was carried out.
